# Multilingualism and Cognitive Impairment: Are they related?

**DOI:** 10.1002/alz70857_107800

**Published:** 2025-12-24

**Authors:** Apurva Mittal, Kavya K Kumar, Jyothi M S Gowda, Aparna N P, Himanshu Joshi, Subhashini K Rangarajan, Preeti Sinha, Mathew Varghese, Ravikesh Tripathi, Sivakumar PT, Ganesan Venkatasubramanian, Paul M. Thompson, John P John

**Affiliations:** ^1^ National Institute of Mental Health and Neurosciences (NIMHANS), Bengaluru, Karnataka, India; ^2^ Multimodal Brain Image Analysis Laboratory (MBIAL), National Institute of Mental Health and Neurosciences (NIMHANS), Bengaluru, Karnataka, India; ^3^ St John's Medical College, Bengaluru, Karnataka, India; ^4^ National Institute of Mental Health and Neurosciences (NIMHANS), Bangalore, Karnataka, India; ^5^ Translational Psychiatry Laboratory, National Institute of Mental Health and Neuro Sciences (NIMHANS), Bengaluru, Karnataka, India; ^6^ Imaging Genetics Center, Mark and Mary Stevens Neuroimaging & Informatics Institute, University of Southern California, Marina del Rey, CA, USA; ^7^ National Institute of Mental Health And Neurosciences, Bengaluru, Karnataka, India; ^8^ Multimodal Brain Image Analysis Laboratory, National Institute of Mental Health and Neurosciences (NIMHANS), Bengaluru, Karnataka, India

## Abstract

**Background:**

Although converging evidence shows multilingualism may improve cognitive reserve and protect against Alzheimer's dementia (AD), results from cross‐sectional and longitudinal studies are inconsistent. We examined whether cognitive performances in AD, Mild Cognitive Impairment (MCI), and healthy elders (HE) differ based on multilingualism (ML) and Language Proficiency (LP).

**Method:**

We carried out this study through the NIH/NIA‐funded R01 project (1R01AG060610) titled “India ENIGMA Initiative for Global Ageing and Mental Health”. ML was measured by evaluating how many languages were known to the participants. They were then grouped into two categories: those who knew three or more languages and those who knew fewer than three. Language proficiency (LP) was measured in three areas—speaking, reading, and writing. In each area, participants were divided into the similar two groups as above (e.g., those who could speak three or more languages vs. fewer than three). We used multivariate analysis to compare several cognitive performance measures such as delayed recall of word list, design construction, story memory test, category and phonemic fluency, between the ML and LP groups. This was done separately for each diagnosis—HE, MCI, and AD. For MCI and AD, we controlled for Clinical Dementia Rating (CDR) score.

**Result:**

85 age matched participants (HE=47, MCI=24, AD=14) were analyzed. ML was significantly different across the HE, MCI, AD (knowing >=3 languages: HE=78.7%, MCI=79.1%, AD=35.71%, χ2=10.75, *p* = 0.005). Further, LP differed significantly ((speaking >=3 languages: HE=72.3%, MCI=73.9%, AD=21.4%, χ2=13.46, *p* = 0.001) or reading >=3 languages: HE=55.3%, MCI=69.6%, AD=14.3%, χ^2^ = 11.03, *p* = 0.004)).

There was a trend towards a significant effect of ML in MCI (Wilks Λ=0.48, F(5,12)=2.55, *p* = 0.08, ηp^2^ = 0.5) in multivariate analysis, in which design construction delayed recall was significantly different (*p* = 0.004, ηp^2^ = 0.4). LP in speaking had a significant effect in MCI (Wilks Λ=0.37, F(5,12)=4.08, *p* = 0.02, ηp^2^ = 0.6) in design construction (*p* = 0.01, ηp^2^ = 0.34) and AD (Wilks Λ=0.05, F(5,3)=10.69, *p* = 0.04, ηp^2^ = 0.9) in delayed recall of word list (*p* = 0.001, ηp^2^ = 0.79).

**Conclusion:**

Knowing multiple languages and having proficiency in speaking and reading seems to be significantly different between the elderly with and without cognitive impairment. ML and LP may modulate various cognitive domains differentially in those with MCI and AD.

